# Uncovering spatial variation in maternal healthcare service use at subnational level in Jimma Zone, Ethiopia

**DOI:** 10.1186/s12913-020-05572-0

**Published:** 2020-07-31

**Authors:** Jaameeta Kurji, Benoit Talbot, Gebeyehu Bulcha, Kunuz Haji Bedru, Sudhakar Morankar, Lakew Abebe Gebretsadik, Muluemebet Abera Wordofa, Vivian Welch, Ronald Labonte, Manisha A. Kulkarni

**Affiliations:** 1 School of Epidemiology and Public Health, University of Ottawa, 600 Peter Morand Crescent, Ottawa, Ontario K1G 5Z3 Canada; 2 Jimma Zone Health Office, Jimma Zone, Oromia Region, Jimma, Ethiopia; 3 Department of Health, Behaviour & Society, Jimma University, Jimma, Ethiopia; 4 Department of Population & Family Health, Jimma University, Jimma, Ethiopia; 5 Centre for Global Health, Bruyere Research Institute, Ottawa, Canada

**Keywords:** Ethiopia, Spatial analysis, Clusters, Maternal health service use, Sub-national data, Equity

## Abstract

**Background:**

Analysis of disaggregated national data suggest uneven access to essential maternal healthcare services within countries. This is of concern as it hinders equitable progress in health outcomes. Mounting an effective response requires identification of subnational areas that may be lagging behind. This paper aims to explore spatial variation in maternal healthcare service use at health centre catchment, village and household levels. Spatial correlations of service use with household wealth and women’s education levels were also assessed.

**Methods:**

Using survey data from 3758 households enrolled in a cluster randomized trial geographical variation in the use of maternity waiting homes (MWH), antenatal care (ANC), delivery care and postnatal care (PNC) was investigated in three districts in Jimma Zone. Correlations of service use with education and wealth levels were also explored among 24 health centre catchment areas using choropleth maps. Global spatial autocorrelation was assessed using Moran’s I. Cluster analyses were performed at village and household levels using Getis Ord Gi* and Kulldorf spatial scan statistics to identify cluster locations.

**Results:**

Significant global spatial autocorrelation was present in ANC use (Moran’s I = 0.15, *p* value = 0.025), delivery care (Moran’s I = 0.17, *p* value = 0.01) and PNC use (Moran’s I = 0.31, *p* value < 0.01), but not MWH use (Moran’s I = -0.005, *p* value = 0.94) suggesting clustering of villages with similarly high (hot spots) and/or low (cold spots) service use. Hot spots were detected in health centre catchments in Gomma district while Kersa district had cold spots. High poverty or low education catchments generally had low levels of service use, but there were exceptions. At village level, hot and cold spots were detected for ANC, delivery care and PNC use. Household-level analyses revealed a primary cluster of elevated MWH-use not detected previously. Further investigation of spatial heterogeneity is warranted.

**Conclusions:**

Sub-national variation in maternal healthcare services exists in Jimma Zone. There was relatively higher poverty and lower education in areas where service use cold spots were identified. Re-directing resources to vulnerable sub-groups and locations lagging behind will be necessary to ensure equitable progress in maternal health.

## Background

Improvements in access to services across the continuum of maternal healthcare have been reported throughout the world but progress has been unequal [1]. In addition to differential successes between countries, uneven access to essential maternal healthcare services within countries have also been revealed [2] using disaggregated national data. In Ethiopia, nationally representative Demographic and Health Survey (DHS) data show markedly different service coverage levels associated with reductions in maternal mortality; in 2016, the proportion of births at health facilities ranged from close to 60% in Tigray and Dire Dawa regions to under 20% in Somali and Affar regions [3].

Of major concern is the fact that women who are the most vulnerable and likely in need of healthcare services may be least likely to access them. Inequity in health arises as a result of “unfair and avoidable” differences [4]. The concept is linked to that of the social determinants of health which recognizes that the conditions in which individuals live shape their ability to access resources and can result in unfair disadvantages thus perpetuating inequalities in health outcomes [5]. Improving equitable outcomes in multiple domains affecting health and human development underlies the 2015 Sustainable Development Goals, including an explicit goal to “reduce inequality between and within countries” (Goal 10), highlighting the value placed on this principle by most of the world’s governments [6].

Inequalities in maternal healthcare service use have been reported across countries in sub-Saharan Africa [7–9]. Education and household wealth are important factors affecting access to services. Several cross-sectional studies in Ethiopia have reported that women with higher levels of education are more likely to have used antenatal care (ANC) [10, 11] and have given birth at a health facility [12–14]. Similar associations have been reported wherein increases in household wealth have corresponded with increased odds of maternal healthcare service use [11, 15].

Decentralization of health service provision and management means that local level policy makers require evidence about service utilization at that level. Spatial analyses present a powerful medium for visually demonstrating service utilization patterns at various geographic levels. Superimposition of data layers can help end users ascertain which areas require attention and identify marginalized populations that require targeted support.

In this paper, we explore spatial variation in the utilization of antenatal, delivery and postnatal care (PNC) services and use of maternity waiting homes (MWH) at health centre catchment and village levels, and assess spatial correlations with household wealth and women’s education levels in three rural districts in Ethiopia.

## Methods

### Study setting

**Fig. 1:**
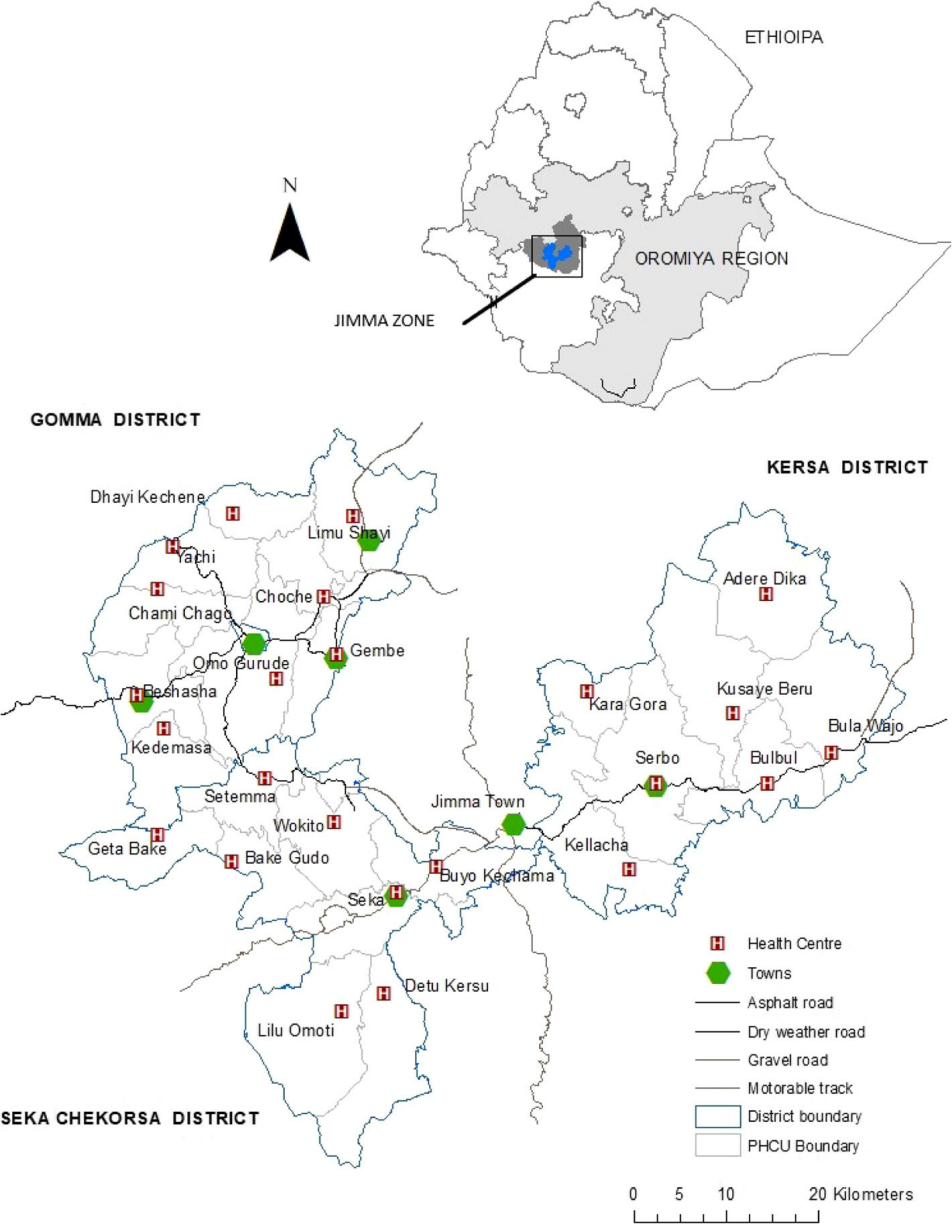
Study area map showing PHCU boundaries and locations of health centres within PHCUs. The figure was generated in ArcMap 10.6.1 using study GPS data shapefiles obtained from the Jimma Zone Health Office

Ethiopia is composed of nine regional states and two city administrations; these are sub-divided into *woredas* (districts) which comprise several *kebeles* (villages). This analysis focuses on three rural *woredas* (Gomma, Seka Chekorsa and Kersa) in Jimma Zone, located in south-western Ethiopia in Oromiya region as shown in Fig. 1. Populations in the *woredas* range from 180,000 to 270,000 in 2016 [16]. Coffee production is an important source of revenue for residents of Gomma while Seka Chekorsa and Kersa residents engage mainly in small-scale cereal production [16]. As part of the tiered health care system in Ethiopia, Jimma Zone has two general hospitals, six district hospitals, 122 health centres and 566 health posts [17]. The lowest health system level functions at the *woreda* level and consists of primary health care units (PHCUs). Each PHCU includes a health centre serving around 25,000 people and community-based health posts responsible for a population of 3000-5000. Regional level data indicate that in 2016, 48.6% of women surveyed in Oromiya Region in the DHS received no antenatal care and just 18.8% of women delivered at a health facility, placing the region among the poorer performing areas in the country [3].

### Data source

Data from a cross-sectional household survey, conducted prior to intervention roll-out (baseline survey) between October 2016 and January 2017 as part of a cluster-randomized controlled trial (ClinicalTrials.gov; NCT03299491), was used for this analysis [18]. Women living in the catchment area of 24 PHCUs within the study’s three districts and who reported a pregnancy outcome (livebirth, stillbirth, miscarriage or spontaneous abortion) within 12 months of the baseline survey were eligible to participate. Sample size (24 PHCU clusters with 160 women each) was calculated based on 80% power to detect an absolute difference of 0.17 in the proportion of institutional births (primary trial outcome) assuming a control arm proportion of 0.4 and using a two-sided alpha of 0.025 to account for two pairwise comparisons. Verbal informed consent was sought from all participants owing to low levels of literacy. Approximately 98.5% (*n* = 3784) of the targeted women were successfully enrolled and interviewed in either Afaan Oromo or Amharic by trained research assistants. Data were collected on sociodemographic characteristics and maternal healthcare service utilization using tablet computers programmed with surveys using Open Data Kit software. GPS locations of households and health centres were also collected on tablet computers; GPS locations were available for 3758 (97.9%) of the enrolled households.

### Variables of interest

The analysis focused on four services across the continuum of maternal healthcare: antenatal care, maternity waiting home use, delivery care at health facilities, and postnatal care. As women’s education levels and household wealth have previously been linked to access inequities [19], we adopted them for our analyses. Operational definitions of all the variables of interest are described in Table 1.

**Table 1:** Operational definitions of analysis variables used to describe maternal healthcare service use among study women

Variable	Definition
Antenatal care	The proportion of women in the PHCU (or *kebele*) who report receiving any antenatal care during their last pregnancy (i.e at least one visit to a health centre or health post where antenatal care services are offered)
Maternity waiting home use	The proportion of women in the PHCU (or *kebele*) who report ever having used an MWH during their previous pregnancies.
Delivery care	The proportion of women in the PHCU (or *kebele*) who report giving birth to their last child at a health facility offering obstetric care (i.e health centre or hospital).
Postnatal care	The proportion of women in the PHCU (or *kebele*) who report receiving any postnatal care following the birth of their last child (i.e at least one postnatal care check-up within 6 weeks of delivery)
Education	The proportion of women in a PHCU (or *kebele*) who report having some formal education (i.e some primary, secondary or higher-level education).
Household wealth	The proportion of households in the PHCU (or *kebele*) who fall within the highest two household income quintiles (i.e fourth and fifth quintiles of household wealth) as determined using an asset-based principal components analysis.

Principal components analysis was used to create the household wealth variable using ownership of assets and animals, utilization of health insurance, presence of electricity supply, type of drinking water source, type of toilet present, and type of materials used for floor construction of the home. Using methods described by Vyas & Kumaranayake [20], socio-economic scores were generated for each household and then categorized into quintiles. The first quintile corresponds to poorest households while the fifth quintile corresponds to the least poor households.

### Exploratory analyses

Frequencies and percentages of education and wealth levels in survey clusters were generated in STATA version 15 as part of descriptive analyses. Differences between survey clusters in the percentages of wealthy and educated households were then compared using a chi square test. *P* values less than 0.05 considered to be statistically significant.

Euclidean distances between households and the health centre within the PHCU catchment were calculated in kilometres for each PHCU using the *Generate Near Table* tool in ArcMap version 10.6.1. Distances were summarized using means, standard deviations and ranges and classified into three categories (< 2 km, 2-5 km and > 5 km) to facilitate comparison between PHCUs using a chi square test.

Geographical variation in maternal healthcare service use was explored at the PHCU-level, the level at which maternal and other primary care services are coordinated and at the *kebele*-level, which is the smallest administrative unit and where health posts, staffed by two health extension workers (HEWs) are located. To this end, the proportion of women who reported using a given service during their last pregnancy within each geographical unit (PHCU or *kebele*) in the study area was calculated. Additionally, household-level binary data was used to explore variation in service use without constraining analyses within administrative boundaries. Instead, a maximum radius of 5 km, at which we hypothesize interpersonal and social factors affecting service use could operate, was used. Other studies in similar settings have estimated a one-hour walking distance to be between 3 km to 5 km depending on the season and terrain [21, 22]. District-level analyses were not feasible given the limited number of districts. At the PHCU-level, choropleth maps were generated; choropleth maps present interval data superimposed over geographic units using colours and symbols to distinguish between intervals [23]. To explore utilization levels among marginalized population sub-groups (women from poor households and women with no education), education and household wealth were symbolized as separate layers and superimposed to visualize correlations. Intervals for all variables were manually created based on what best suited the distribution of each variable, meaning that only qualitative comparisons can be made between services as intervals differ between services. Each maternal health care service was assigned a different colour with darker shades indicating higher extents of service. Wealth and education indicators are overlayed using proportional symbols where larger circles correspond to higher levels. Administrative boundaries for PHCUs were created by dissolving boundaries of villages known to fall within the catchment area of the PHCU using the Dissolve tool in ArcMap. PHCU size varied from 53 km^2^ (sq km) to 186 sq. km; the mean area was 108.5 sq. km (standard deviation 39.6 sq. km).

To examine variation in service use at the *kebele* level, the presence of spatial autocorrelation for each service was first examined using the global Moran’s I statistic. The results of this test indicate whether or not the spatial patterns observed in the data are random by looking at how each *kebele* deviates from the mean among neighbouring *kebeles*. Statistically significant and positive Moran’s I indices indicate the presence of clustering (i.e a high degree of similarity in levels of service use between neighbouring *kebeles*) while negative values specify dispersion (i.e neighbouring *kebeles* have dissimilar values) [24]. An inverse-distance-based spatial relationship conceptualization was selected for the process; this means that *kebeles* outside the threshold distance are not included in the computations. The threshold distance at which every *kebele* had at least one neighbouring *kebele* was determined to be 7.6 km. Of the 100 *kebeles* present in the study area, data were available for 96. An average of 48 women were enrolled per *kebele*. One *kebele* with less than five women enrolled was excluded as this number was considered to be too low for the analysis.

The next step was to pinpoint the location of clusters. The *Optimized Hot Spot Analysis* tool in ArcMap was used to identify where clustering was occurring. This tool relies on the Getis Ord Gi* spatial statistic to uncover statistically significant hot spots (clusters of high service use) and cold spots (clusters of low service use). Clusters are considered statistically significant only if they are surrounded by similarly high or low values [25]. The optimal fixed distance band is determined by the tool for each service outcome by determining at what distance clustering is maximized. This was found to be 10 km for ANC, 7 km for MWH use and 19 km for delivery care. For PNC, a threshold distance of 7 km was used as no optimal distance was found using the clustering intensity method. The resulting output is a map of statistically significant hot (red) and cold (blue) spots presented at 99% (+/− 3 Gi* values), 95% (+/− 2 Gi* values) and 90% significance (+/− 1 Gi* values) and corrected for multiple testing.

Finally, household-level data on women’s reported service use were analysed using the Kulldorf spatial scan statistic in SaTScan 9.6. This spatial tool functions by running circular “scanning windows” of multiple sizes across the study space. The events observed within the window are compared to those expected under the null hypothesis of no difference inside and outside the window; a relative risk (RR) is generated which represents the ratio of events observed and expected within the window to those observed and expected in the study area. The formula used in SaTScan [26] is:

$$ \mathrm{RR}=\frac{\mathrm{c}/\mathrm{E}\left[\mathrm{c}\right]}{\left(\mathrm{C}\hbox{-} \mathrm{c}\right)/\left(\mathrm{C}\hbox{-} \mathrm{E}\left[\mathrm{c}\right]\right)} $$

where c is the observed number of events within a potential cluster (window).

E[c] is the expected number of events within a potential cluster (window).

C is the total number of events in the dataset.

The overall proportion of events in the study area were 84.3% for ANC, 6.6% for MWH use, 48.5% for delivery care and 39.0% for PNC and are used to calculate the expected number of events within a cluster (E[c]). A relative risk greater than one would suggest proportion observed within the window is higher than expected while relative risks less than one suggest observed proportions are lower than expected. A Bernoulli model was used due to the binary nature of the outcomes and the window radius set to a maximum of 5 km. The most likely cluster is identified as the window with the maximum likelihood, meaning it was least likely to have occurred by chance. Monte Carlo hypothesis testing is used to generate *p*-values where ranks of maximum likelihood of the observed dataset is compared to random datasets. Secondary clusters are also identified and ranked according to the Likelihood Ratio Test statistic [26].

## Results

### Characteristics of PHCUs

The size of PHCUs varied, with total number of households ranging from 2509 households to 11,791 households (Table 2). There were also statistically significant differences (p-value < 0.001) between the PHCUs in the percentage of educated women and wealthy (least poor) households. PHCUs with the highest proportion of educated women (69%) and wealthy households (81%) were located in Gomma district. Kusaye Beru in Kersa district had the lowest proportion of educated women (25%) while the PHCU with the lowest percentage of wealthy households was located in Seka Chekorsa (7%).

**Table 2:** Characteristics of PHCUs and sampled clusters within Gomma, Seka Chekorsa and Kersa districts

PHCU by district	PHCU characteristics^1^			Cluster characteristics						
	Total households	Total women	Health posts	n	Educated households n (%)^2^		***p*** value	Wealthy households^3^ n (%)		***p*** value
**Gomma**						< 0.001			< 0.001	
Beshasha	7556	8026	5	154	92	59.7		124	80.5	
Chami Chago	5808	6170	4	189	90	47.6		139	73.5	
Choche	5889	6256	4	159	109	68.6		115	72.3	
Gembe	5242	5568	4	130	74	56.9		84	64.6	
Dhayi Kechene	2509	2665	2	159	85	53.5		57	35.9	
Kedemasa	3980	4228	3	165	59	35.8		52	31.5	
Limu Shayi	6696	7113	5	158	75	47.5		99	62.7	
Omo Gurude	11,791	12,525	7	136	83	61.0		89	65.4	
Yachi	4921	5227	3	152	74	48.7		113	74.3	
**Kersa**										
Kusaye Beru	5581	5928	5	111	28	25.2		15	13.5	
Bulbul	4332	4602	4	116	44	37.9		34	29.3	
Adere Dika	4813	5113	3	161	42	26.1		39	24.2	
Kara Gora	3921	4165	3	161	42	26.1		29	18.0	
Kellacha	8906	9460	6	153	56	36.6		45	29.6	
Serbo	10,666	11,330	7	242	96	39.7		57	23.6	
Bula Wajo	5636	5987	3	166	48	28.9		43	25.9	
**Seka Chekorsa**										
Bake Gudo	4676	4967	4	159	64	40.3		45	28.3	
Detu Kersu	5990	6363	4	158	78	49.4		11	7.0	
Geta Bake	3483	3699	3	160	81	50.6		38	23.8	
Buyo Kechama	4863	5166	5	134	56	41.8		48	35.8	
Lilu Omoti	9525	10,118	6	152	63	41.5		47	30.9	
Seka	5119	5438	6	222	98	44.1		61	27.5	
Setemma	7118	7561	4	160	89	55.6		76	47.5	
Wokito	6109	6489	4	127	57	44.9		53	41.7	
**Total**	**145,130**	**154,163**	**104**	**3784**	**1683**	**44.5**		**1513**	**40.0**	

^1^Obtained from the Jimma Zone Health Office records

^2^Proportion of households within the cluster where women report having received some level of education (i.e primary, secondary or higher) obtained from the baseline household survey conducted in 2016/2017

^3^Proportion of households within the cluster that fall in two least poor quintiles (4th and 5th) based on the asset-based wealth index scores obtained from the baseline household survey conducted in 2016/2017

**Fig. 2:**
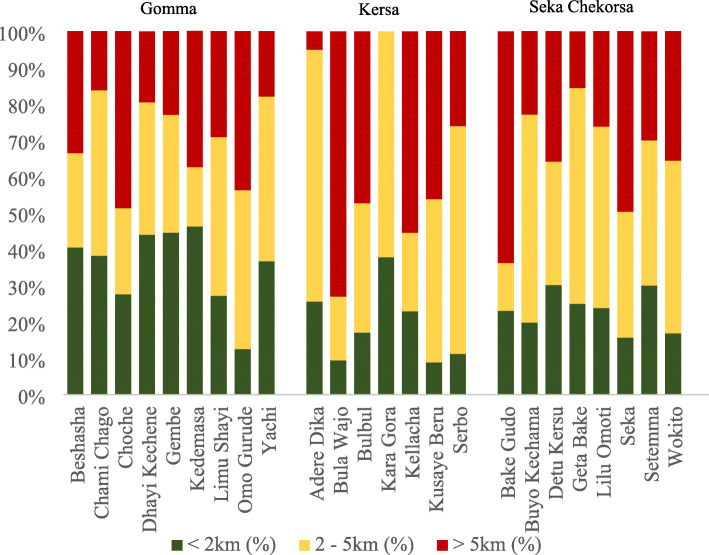
Percentages of households within 2 km, between 2 and 5 km and more than 5 km from health centre

Households were located an average of 4.2 km from health centres within their PHCUs with straight line distances ranging from just 100 m to 18 km. There was a statistically significant difference in distances between the PHCUs (*p* value> 0.001) (data not shown). Twelve PHCUs had over 30% of their households located more than five kilometres from the catchment health centre; Bula Wajo (73%), Bake Gudo (64%) and Kellacha (55%) had more than half the population located greater than five kilometres from the catchment health centre (Fig. 2). The majority of women (*n* = 3167, 87%) reported reaching health facilities by foot.

### Variation in service use at PHCU level and correlation with household wealth and education

**Fig. 3:**
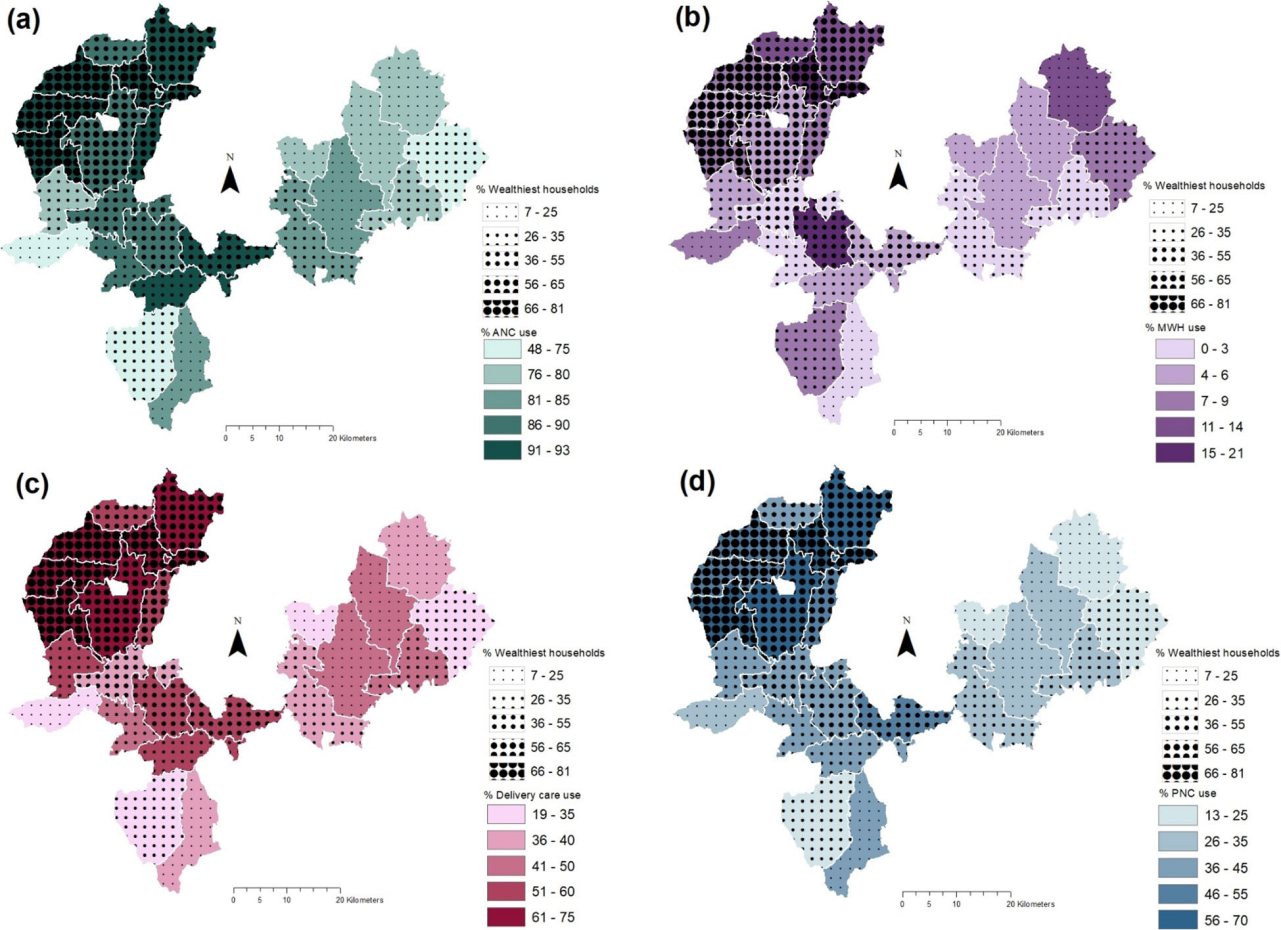
Choropleth maps highlighting correlation between household wealth and (**a**) ANC use (**b**) MWH use (**c**) Delivery care and (**d**) PNC use at PHCU-level

As shown in Fig. 3a, ANC use was generally higher among PHCUs with higher levels of wealthy households. PHCUs with high ANC use were mainly concentrated in the north-western part of the study area in Gomma district and parts of Seka Chekorsa. PHCUs with ANC use reported to be above 90% also had more than 60% of households in the upper two (least poor) wealth quintiles. Buyo Kechama and Seka were the exceptions with over 90% ANC use but having about half as many wealthy households (34 and 28% respectively) as similar performing PHCUs. Delivery care use (Fig. 3c) had similar correlations to wealth as ANC use, with wealthier PHCUs displaying higher relative use of services and poorer PHCUs exhibiting lower utilization levels. However, there were a few notable exceptions where service use did not correspond to relative wealth. For example, Kusaye Beru (delivery care:43%) which had service use levels as low as that found in Bulbul (delivery care:41%) and Bake Gudo (delivery care:43%) but had half as many least poor households (least poor:14% vs. 29 and 28% respectively). Similarly, Setemma with almost half the households belonging to the wealthiest quintiles had delivery care use levels (delivery care:38%) that matched Detu Kersu (delivery care: 36%) where only 7% of households are wealthy. The correlation between lower wealth levels and low service use was particularly evident with PNC use patterns (Fig. 3d) in PHCUs in Kersa district; 30% or less of the households were in the least poor groups and PNC use was well below average, ranging from 13 to 32%.

Correlation between household wealth and MWH use was more variable than for the other three services (Fig. 3b); several PHCUs with more than 50% of households in the least poor group such as Chami Chago or Omo Gurude had utilization levels between 4 and 6% while PHCUs such as Adere Dika (MWH use: 13%) and Dhayi Kechene (MWH use: 14%) that were comparatively poorer had above average levels of use. However, PHCUs with under 30% of least poor households such as Bake Gudo or Detu Kersu had lower than average MWH use (< 1%).

**Fig. 4:**
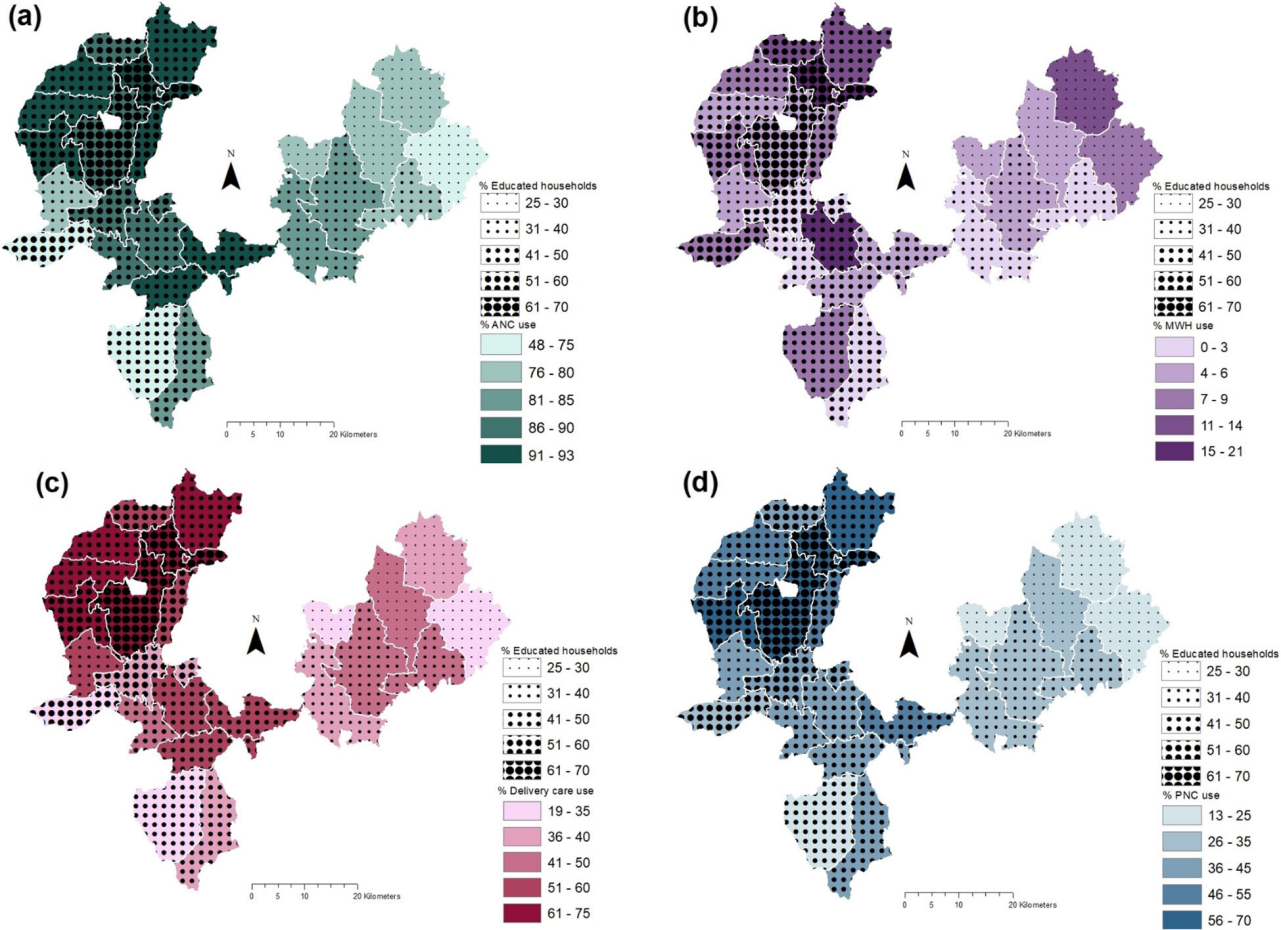
Choropleth maps highlighting correlation between women’s education and and (**a**) ANC use (**b**) MWH use (**c**) Delivery care and (**d**) PNC use at PHCU-level

A trend similar to that observed with wealth was noted between service use and women’s education as displayed in Fig. 4. PHCUs in north-western segment of the study area generally had higher utilization levels of ANC, delivery care and PNC and education levels than the north-eastern part. In fact, the PHCUs with the highest utilization of services were consistently located in Gomma and the northern sector of Seka Chekorsa, near Jimma town, and also had relatively higher levels of women who reported some level of education. Once again, however, there were exceptions. For example, Geta Bake registered lower levels of service use despite having over 50% education levels which was higher than some of the better performing PHCUs north of it or indeed neighbouring Bake Gudo.

### Spatial clustering in maternal health care service use at village level

The results of the global Moran’s I tests pointed to the presence of spatial autocorrelation in the study area with respect to ANC use (Moran’s I = 0.15, *p* value = 0.025), delivery care (Moran’s I = 0.17, *p* value = 0.01), and postnatal care (Moran’s I = 0.31, *p* value < 0.01), but not MWH use (Moran’s I = -0.005, *p* value = 0.94). This means that there is clustering of *kebeles* with similarly high and/or low service utilization levels.

**Fig. 5:**
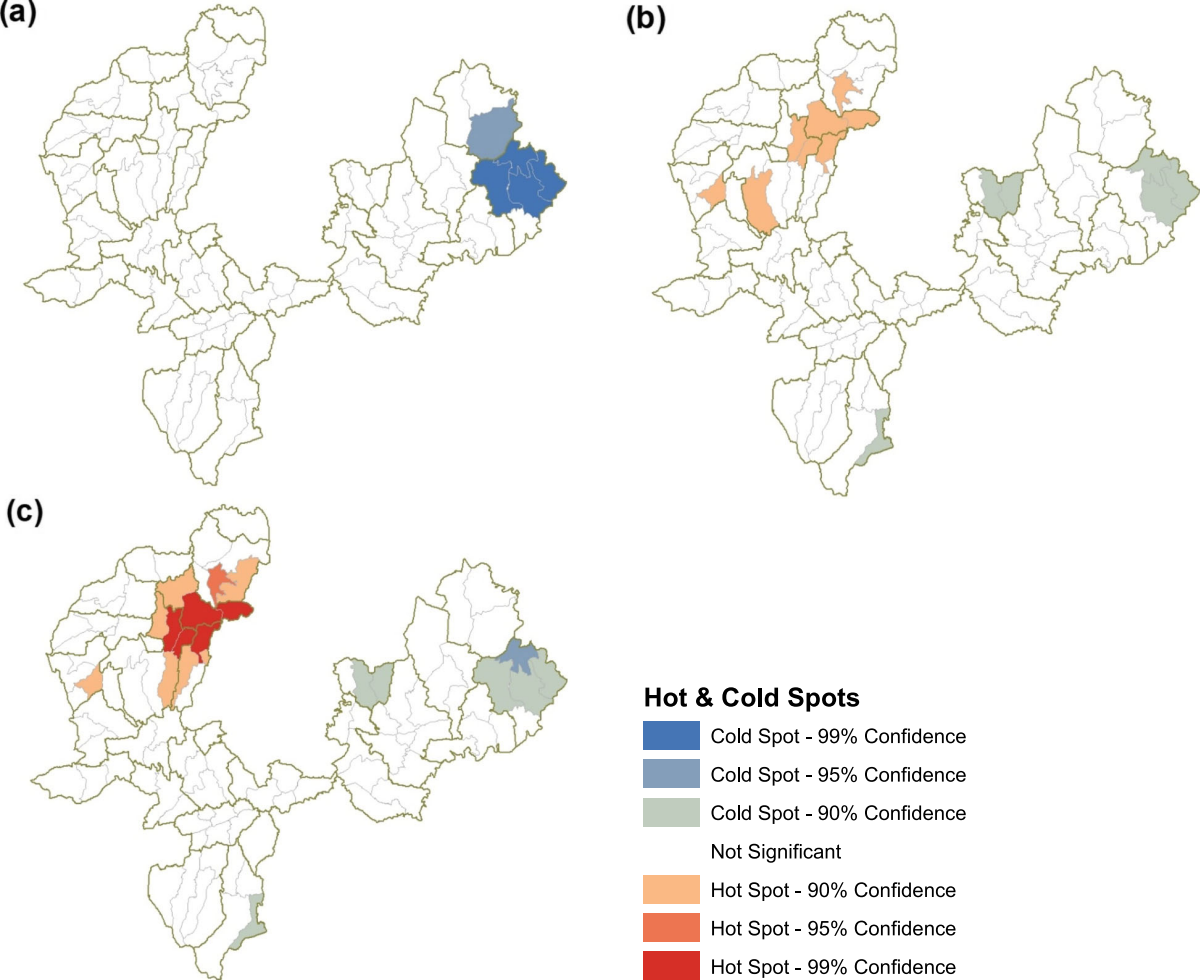
Hot and cold spots at kebele-level of (**a**) ANC use (**b**) Delivery care use (**c**) PNC use in study districts

The locations of service utilization clusters identified using the Getis Ord Gi* spatial statistic are displayed in the panel of maps in Fig. 5. Since no spatial autocorrelation was detected in MWH use, cluster detection was limited to the three other services. For ANC use, four *kebeles* in Bula Wajo PHCU and one in Adere Dika were found to be statistically significant cold spots (Fig. 5a). This means that although there may have been other *kebeles* with similarly low ANC utilization levels in the study area, these four had low levels of use and were surrounded by similarly low performing *kebeles*. For delivery care, eight hot and six cold spots were found; hot spots were located in *kebeles* in Gomma PHCUs and cold spots in Kersa PHCUs (Fig. 5b). Hot spot *kebeles* all recorded delivery care use well above the study area average of 49% and all except two *kebeles* had utilization levels higher than the Gomma district level use (64%). Among cold spot *kebeles*, Sinkulle and Dogoso in Bula Wajo PHCU had very low delivery care use reported (7 and 2% respectively).

*Kebele* clusters for PNC use were almost identical to delivery care except that there were additional hot spots detected in Limu Shayi, Omo Gurude, Gembe and Choche PHCUs. Among the 13 hot spots identified, eight had over 65% PNC use with 82% of women in Bulbulo reporting PNC use after the birth of their last child. Cold spots exhibited PNC use levels comparable to delivery care; once again *kebeles* in Bula Wajo PHCU had exceptionally low levels of PNC use ranging from 3 to 5%. The cold spot in Kora Wacho in Seka Chekorsa had PNC use at 10%.

**Fig. 6:**
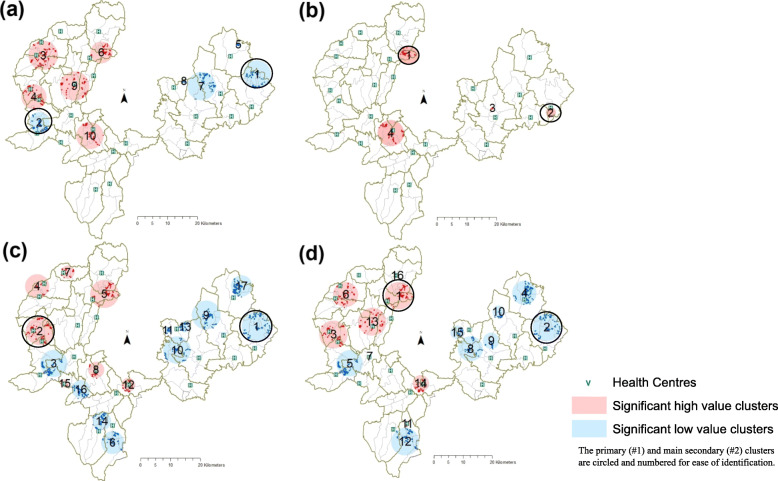
Clusters within *kebeles* of (**a**) ANC use (**b**) MWH use (**c**) Delivery care use (**d**) PNC use in study districts

Locations of statistically significant clusters detected for each service using the Kulldorf spatial scan statistic are shown in Fig. 6. Details about the primary cluster (most likely cluster) and main secondary cluster are displayed in Table 3.

**Table 3:** Primary and main secondary household-level clusters of service use detected using Kulldorf spatial scan statistic

	Cluster population	Observed use within cluster (c)	Expected use within cluster (E [c])	Relative Risk (RR)	***p***-value
**Primary clusters**					
Antenatal care	81	12	68	0.17	< 0.0001
Maternity waiting homes	65	22	4	5.56	< 0.0001
Delivery care	120	11	58	0.18	< 0.0001
Postnatal care	133	102	52	2.04	< 0.0001
**Main secondary cluster**					
Antenatal care	95	43	81	0.53	< 0.0001
Maternity waiting homes	12	10	1	12.16	< 0.0001
Delivery care	216	170	104	1.69	< 0.0001
Postnatal care	116	7	45	0.15	< 0.0001

A primary cluster of households was found from *kebeles* in Bula Wajo PHCU north of the health centre which exhibited lower than expected ANC use (Relative Risk [RR] = 0.17, *p* < 0.0001). The main secondary cluster for ANC use was located between Kedemasa and Geta Bake PHCUs close to the Geta Bake health centre (RR = 0.53, *p* < 0.0001). A total of ten statistically significant clusters were detected for ANC use.

Elevated MWH use was also found, with a total of five high value use clusters detected. The primary cluster was located among households in Dinu and Tesso Sadecha *kebeles* in Choche and Limu Shayi PHCUs (RR = 5.6, *p* < 0.0001). The main secondary cluster also of elevated MWH use was situated near Bula Wajo health centre (RR = 12.16, *p* < 0.0001).

The primary cluster detected for delivery care was a cold spot from households in low performing *kebeles* in Bula Wajo PHCU (RR = 0.18, *p* < 0.0001). Nine additional secondary cold spots and seven hot spots of delivery care were also found. The main secondary cluster of elevated delivery care use was found among households around the Beshasha and Kedemasa health centres in Gomma district (RR = 1.69, *p* < 0.0001).

Finally, 16 statistically significant clusters were found for PNC use. The most likely clusters of households (primary cluster) was found in *kebeles* in Limu Shayi, Choche and Gembe PHCUs which exhibited higher than expected PNC use (RR = 2.04, *p* < 0.0001). The main secondary cluster was of lower use situated in Bula Wajo PHCU (RR = 0.15, *p* < 0.0001). Additional information on all secondary clusters can be found in the Supplementary table.

## Discussion

This study demonstrated the existence of significant variation in levels of maternal health care service use at PHCU and *kebele* levels in Jimma Zone. It also found that PHCU-level variations in level of household wealth and women’s education generally correlated with service utilization trends. However, several exceptions to this trend were noted where low utilization was registered in some locations with higher education or wealth or vice versa. This points to the need to explore spatial heterogeneity, i.e. the existence of regionally specific associations, to determine the relative importance of wealth and education as well as other factors in determining service use. Although several determinants of service use have been reported in the literature, including women’s education [27–29], place of residence [29, 30], or household wealth [29, 31], these studies generally rely on statistical models that generate parameter estimates that are constant over space; this approach assumes that the influence of determinants is the same at every location [32]. However, this exploratory study suggests that this may not always be the case and warrants further investigation.

Different patterns between service use and household wealth or women’s education were also noted between PHCUs depending on the type of service. Whereas for ANC, delivery care and PNC the correspondence between service use and wealth or education was roughly similar, the effect of wealth on MWH use appeared to be less straight-forward. This may be because MWH use is moderated by need which is dependent on distance and access to transport. It is reasonable to assume that women living close to health facilities or who are able to easily travel (such as women with more resources) would opt to go to a health facility when in labour obviating the need to stay at an MWH. Utilization may also be influenced by other factors such as type of occupation or access to social support [33]. This may be the case in Omo Gurude for instance, where despite having a higher percentage of wealthy (65%) and educated (61%) households and almost 45% of the population living more than 5 km from a health centre, MWH use was slightly less than average. The primary clusters detected for ANC, delivery care and PNC, which were all cold spots, were located some distance from the PHCU health centre suggesting distance may play a role in unequal access to services, which should be explored further.

While no causal inferences about associations between service use and wealth or education can be made through this exploratory analysis, this work suggests that marginalized groups may exist in areas of low maternal health care service use in this rural area of Ethiopia. Similar results have been reported in neighbouring Kenya wherein counties with high rates of poverty and illiteracy had much lower levels of skilled birth attendance than counties with better socioeconomic indicators. Moreover, clear geographical differences in skilled birth attendance and child immunizations were noted on choropleth maps generated by the authors [34].

The results from both global and local spatial statistics suggest that underlying spatial processes may be influencing maternal healthcare service utilization in the study area. The location-specific observed patterns indicate that similar utilization tendencies were located close together. Several studies have reported differential utilization according to place of residence with rural areas falling short in antenatal [35], delivery [31, 36, 37] and postnatal care [38] use compared to urban areas. PHCU-level variation may be reflective of differences in service delivery and quality. Qualitative evidence from Ethiopia suggested contrasting levels of collaboration between health extension workers (HEWs) and health centre staff, variable coordination between higher-level administrative bodies (district, zone and region) and use of available data for decision making at district level [39] which may contribute to differences observed in service use.

Investigating sub-national variation in health outcomes and access to health services has become a priority with the realization that equitable progress will only be achieved if populations that are under-served are identified and supported. Moreover, ensuring that maternal health services are able to “respond to local specificities of need …” is critical for ensuring equitably improved maternal health outcomes [40]. As a cross-sectional survey conducted in two urban slums in Lagos, Nigeria (that found maternal mortality within neglected neighbourhoods to be almost double the state average) concluded, the use of sub-national data is urgently needed to enable targeted support based on identified gaps in coverage and access [41].

The presence of spatial processes driving utilization at village level suggests that factors affecting access to maternal health care may operate at these levels and may require more focused community-level interventions. The Ethiopian health system is well-positioned to mount responses at this level via its network of community-based HEWs who are mandated to engage in health promotion and disease prevention activities. HEWs play an active role in improving access to maternal health care services through community education and provision of referrals. Correlations between HEW outreach activities and improvements in antenatal and postnatal care use trends have been demonstrated at village level [42]; home-visits conducted by HEWs have also been reported to increase the odds of ANC use and facility deliveries in Ethiopia [43]. HEWs also work closely with members of the Women’s and Men’s Development Army which have extensive reach at the village level through the model family platform. Model families are households that have successfully implemented changes to their households to improve family health and sanitation; they are expected act as positive influences at the neighbourhood level by encouraging similar change in others [44].

### Study limitations

A common concern with spatially aggregated data is ecological bias which arises when associations found at group-level do not necessarily apply at the individual level, referred to as ecological fallacy [45]. However, household wealth and women’s education have been shown to be important determinants of maternal health care service use at the individual level. What remains to be investigated is the location-specific variability in the relative importance of these equity dimensions and other determinants of service use. These exploratory analyses suggest that there is some spatial non-stationarity in these outcomes which can be assessed using local modelling techniques such as geographically weighted regressions. Future work aiming to investigate what factors drive heterogeneity of service use at sub-national level should account for the potential sensitivity of wealth indexes to the items included in their construction [46].

Euclidean distances were calculated which, while being the simplest way to estimate distances, are often criticized for not accounting for geographic context such as terrain or the availability of roads. Despite this, Euclidean distances have been shown to perform comparatively well; they are proposed to be suitable for low resource settings where road-networks and land-cover data needed for cost-distances, are not easily available [47].

In this exploratory analysis, which aimed to uncover spatial variation in service use by examining sub-national data, facility “by-passing” was not considered. “By-passing” is where women seek care for facilities other than the one closest to them [48]. While this does not affect PHCU-level estimates of service use or change areas with hot/cold spots, this does have implications for future work that aims to investigate location-specific determinants of use. Additionally, the dynamic nature of catchment areas in the context of a growing population, means that spatial patterns may also change with time. The introduction of a new health facility offering maternal health care that brings services closer to women may decrease cold spots. This highlights the need to integrate spatial analyses into routine health service monitoring to make it an effective decision-making tool for policymakers.

The use of community-based survey data rather than medical records minimizes the presence of selection bias which arises when analyses focus on those already able to access health services. Despite this, findings of this study can only be generalized to primarily rural, low-resource settings similar to the study area.

## Conclusions

Mapping of core maternal health care service use indicators can serve as a “decision-making tool” and encourage “social accountability” [49]. This work demonstrated the existence of variation in utilization levels of maternal healthcare services at a sub-national level in rural south-western Ethiopia. It further highlighted that variation is service-dependent. More importantly, these sub-national differences closely reflected differences in poverty and women’s education levels which have been found be linked to social inequities. In order to ensure equitable progress in health improvements across all segments of the population and to make effective use of limited resources, the integration of sub-national indicator mapping into routine health system performance monitoring systems may be helpful.

## Supplementary information

**Additional file 1: Supplementary Tables.** Secondary clusters

## Data Availability

Data used for this analysis will be provided by the authors upon reasonable request. The corresponding author may be contacted via email.

## Abbreviations

ANC: Antenatal care
DHS: Demographic & Health Survey
HEW: Health extension worker
MWH: Maternity waiting home
PHCU: Primary healthcare unit
PNC: Postnatal care
