# Supplementary material for: Uncovering spatial variation in maternal healthcare service use at subnational level in Jimma Zone, Ethiopia
Source: BMC Health Serv Res. 2020 Jul 31;20:703. doi: 10.1186/s12913-020-05572-0 (PMC7394677; doi:10.1186/s12913-020-05572-0)
Supplement: Supplementary file 1 — Additional file 1: Supplementary Tables. Secondary clusters [file 12913_2020_5572_MOESM1_ESM.docx]

**Supplementary Tables.** Secondary clusters

| Cluster | Population | Observed use | Expected use | Relative Risk | p-value |
| --- | --- | --- | --- | --- | --- |
| **Antenatal care** | | | | | |
| 3 | 267 | 256 | 225 | 1.15 | <0.001 |
| 4 | 166 | 163 | 140 | 1.17 | <0.001 |
| 5 | 15 | 3 | 13 | 0.24 | <0.001 |
| 6 | 85 | 85 | 72 | 1.19 | <0.01 |
| 7 | 79 | 47 | 67 | 0.70 | <0.01 |
| 8 | 15 | 4 | 13 | 0.32 | <0.05 |
| 9 | 108 | 106 | 91 | 1.17 | <0.05 |
| 10 | 69 | 69 | 58 | 1.19 | <0.05 |
| **Maternity waiting homes** | | | | | |
| 3 | 6 | 6 | 1 | 15.58 | <0.001 |
| 4 | 87 | 22 | 6 | 4.13 | <0.001 |
| 5 | 112 | 23 | 7 | 3.34 | <0.05 |
| **Delivery care** | | | | | |
| 3 | 131 | 19 | 64 | 0.29 | <0.001 |
| 4 | 138 | 113 | 67 | 1.74 | <0.001 |
| 5 | 133 | 109 | 65 | 1.74 | <0.001 |
| 6 | 62 | 5 | 30 | 0.16 | <0.001 |
| 7 | 59 | 53 | 29 | 1.88 | <0.001 |
| 8 | 40 | 38 | 19 | 1.98 | <0.0001 |
| 9 | 78 | 12 | 38 | 0.31 | <0.0001 |
| 10 | 58 | 7 | 28 | 0.25 | <0.0001 |
| 11 | 24 | 0 | 12 | 0 | <0.001 |
| 12 | 43 | 38 | 21 | 1.84 | <0.01 |
| 13 | 63 | 11 | 31 | 0.36 | <0.01 |
| 14 | 69 | 13 | 33 | 0.38 | <0.01 |
| 15 | 32 | 29 | 16 | 1.88 | <0.01 |
| 16 | 92 | 22 | 45 | 0.49 | <0.05 |
| 17 | 110 | 29 | 53 | 0.54 | <0.05 |
| **Postnatal care** | | | | | |
| 3 | 180 | 123 | 70 | 1.82 | <0.0001 |
| 4 | 146 | 18 | 57 | 0.31 | <0.0001 |
| 5 | 131 | 17 | 51 | 0.32 | <0.0001 |
| 6 | 221 | 132 | 86 | 1.58 | <0.0001 |
| 7 | 18 | 18 | 7 | 2.58 | <0.001 |
| 8 | 48 | 2 | 9 | 0.11 | <0.001 |
| 9 | 39 | 1 | 15 | 0.07 | <0.01 |
| 10 | 30 | 0 | 12 | 0 | <0.01 |
| 11 | 31 | 26 | 12 | 2.17 | <0.01 |
| 12 | 97 | 15 | 38 | 0.39 | <0.01 |
| 13 | 92 | 59 | 26 | 1.67 | <0.05 |
| 14 | 58 | 41 | 23 | 1.84 | <0.05 |
| 15 | 32 | 1 | 13 | 0.08 | <0.05 |
| 16 | 20 | 18 | 8 | 2.32 | <0.05 |
